# The lived experience of chronic pain and dyskinesia in children and adolescents with cerebral palsy

**DOI:** 10.1186/s12887-020-2011-8

**Published:** 2020-03-17

**Authors:** Clare T. McKinnon, Jennifer H. White, Prue E. Morgan, Giuliana C. Antolovich, Catherine H. Clancy, Michael C. Fahey, Adrienne R. Harvey

**Affiliations:** 1 Department of Physiotherapy, Monash University, PO Box 527, Frankston, 3199 Victoria Australia; 2 Neurodisability & Rehabilitation, Murdoch Children’s Research Institute, 50 Flemington Rd, Parkville, 3052 Victoria Australia; 3 Melbourne Ageing Research, National Aging Research Institute, PO Box 2127, Royal Melbourne Hospital, Parville, 3050 Victoria Australia; 4 Neurodevelopment & Disability, The Royal Children’s Hospital Melbourne, 50 Flemington Rd, Parkville, 3052 Victoria Australia; 5 Victorian Peadiatric Rehabilitation Service, Monash Children’s Hospital, 246 Clayton Rd, Clayton, 3168 Victoria Australia

**Keywords:** Cerebral palsy, Dyskinesia, Pain, Children

## Abstract

**Background:**

To explore the lived experience of chronic pain and dyskinesia in children and adolescents with cerebral palsy.

**Methods:**

A convergent parallel mixed methods design was undertaken. First, a quantitative cross-sectional study of participants able to self-report their quality of life was undertaken. This study characterised pain chronicity, intensity, body locations, and quality of life. Second, semi-structured interviews were undertaken with a subset of children and adolescents experiencing chronic pain.

**Results:**

Twenty-five children and adolescents took part in the cross-sectional study, 23 of whom experienced chronic pain and 13 of moderate intensity. Pain was often located in multiple bodily regions (6/21), with no trends in quality of life outcomes detected. Eight participated in semi-structured interviews, which identified three key themes including ‘lives embedded with dyskinesia’, ‘real world challenges of chronic pain’, and ‘still learning strategies to manage their pain and dyskinesia’.

**Conclusions:**

A high proportion of children and adolescents with cerebral palsy and dyskinesia who were able to self-report experienced chronic pain. The physical and emotional impacts of living with chronic pain and dyskinesia existed along a spectrum, from those with lesser to greater extent of their impacts. Children and adolescents may benefit from targeted chronic pain education and management within bio-psychosocial models.

## Background

Cerebral Palsy (CP) is a lifelong heterogeneous disorder classified according to the predominant neuro-motor subtype as spastic, dyskinetic, or ataxic, with prevalence across subtypes 85, 7, and 3% respectively [1–3]. Although predominant dyskinetic subtypes represent a small proportion of the total CP population, these children often experience significant functional limitations and medical comorbidities, including pain [4–6]. Pain in this population is complex to diagnose and difficult to manage, influenced by a range of cognitive, affective, and behavioural mechanisms experienced within the context of home and school environments [7, 8]. Establishing accurate pain prevalence estimates in children with CP is problematic due to differences in methodologies used across studies, with a recent systematic review identifying pain prevalence to vary widely from 14 to 76% in children and young adults with CP [9]. In broader CP populations (across all sub-types), pain is reported to be more common in adolescents compared to younger children, females, and those with severe motor impairment, leading to reduced quality of life [9]. However, little work has explored real world experiences of dyskinesia and chronic pain amongst children and adolescents with CP.

Dyskinesia refers to involuntary, uncontrolled, and recurring movements with fluctuating muscle tone, further differentiated as dystonia or choreo-athetosis [1, 2]. Some children with CP present with both dyskinesia and spasticity described to have a ‘mixed’ motor-type [2]. In children with dyskinesia, acute and chronic pain may be experienced due to non-invasive (e.g. physiotherapy) and invasive (e.g. surgery) interventional management, general health conditions (e.g. headaches, period pain), and other CP related comorbidities [10, 11]. CP related comorbidities are characteristically high in predominant dyskinetic subtypes, and may include scoliosis, hip displacement, muscle contracture, gastrointestinal dysfunction, and the dyskinetic movement disorder itself [10, 12, 13]. These multifactorial causes of pain over childhood add further difficulty to pain management in this complex population of children and adolescents with neuro-disability.

Accessing the voice of children and adolescents with CP who can self-report is a critical source of non-biased information about pain perceptions and experiences within a demographic where proxy pain reporting is often typical [14]. While some children with limited cognitive capacity remain dependent on proxy pain reports, others may develop the capacity to self-report with increasing age, education, therapeutic interventions, and access to augmentative and alternative communication (AAC). Increasing access to AAC means that many children with dyskinetic CP are capable of self-reporting despite high levels of motor and speech impairment [15]. A recent Australian cohort study reflected the unique physical context of children with predominant dyskinetic CP. In that study 55% of study participants were able to effectively communicate, while 70% had severe motor impairment and 71% had speech that was either unclear, not usually understandable, or not understandable at all [16]. Despite growing ability to access AAC in this population, pain communication remains complex. Pain communication is a process that relies on observers (e.g. parent, clinician, school teacher) understanding the feelings, thoughts, and expressions of the child experiencing pain and for that observer to respond appropriately [17]. This interactive process is particularly complicated for those reliant on AAC, where reliability and effectiveness of their pain communication may alter with different observers, levels of pain severity, fatigue, types of AAC, and use of communication partners [18, 19]. A communication partner describes a person who acts as an intermediary between an AAC user and an observer less familiar with a particular communication mode or strategy [19]. Communication partners are proposed to help AAC users share perspectives by enhancing clarity and efficiency of their communication, noted to be of particular benefit in medical settings where communication barriers are identified to exist (e.g. time constraints, health professional skills) [18, 20]. The unique physical context of children with dyskinetic CP is likely to complicate pain management, making them vulnerable to not having their voices heard and an important group to learn more from their experiences.

The objective of this mixed methods study was to explore the lived experiences of chronic pain and dyskinesia through the unbiased lens of children and adolescents with CP able to communicate experiences by either verbalizing or using AAC.

## Methods

A convergent parallel mixed methods design was employed [21]. Firstly, a quantitative prospective cross-sectional study was undertaken of children and adolescents able to self-report their quality of life, with a subset of children who had chronic pain then taking part in qualitative interviews. The study took place in the outpatient departments of The Royal Children’s Hospital and Monash Children’s Hospital, both Australian tertiary hospitals. Written informed consent was obtained from carers for participation, with children and adolescents providing assent for their involvement. Data was collected between January 2018 and March 2019, with ethics approval from The Royal Children’s Hospital, Monash University, and Monash Children’s Hospital Ethics Committees (HREC/17/RCHM/359).

### Quantitative (cross-sectional)

As part of a larger study (not yet published), a cross-sectional study was undertaken capturing self-report data of quality of life and pain amongst children and adolescents with CP and dyskinesia. This study aimed to characterize chronic pain proportions, pain’s influence over quality of life, body locations of pain, and pain intensity.

### Qualitative (interviews)

A subset of children and adolescents who completed the cross-sectional study and reported experiencing chronic pain, were invited to participate in a single, semi-structured, qualitative interview. The aim of the semi-structured interviews was to undertake an in-depth exploration of the impact of living with both chronic pain and dyskinesia through the unique lens of those able to self-report. An interpretative phenomenology analysis (IPA) approach was used to allow research participants to narrate their own research findings and for better understanding of complex, ambiguous, and emotionally laden experiences of chronic pain and dyskinesia [22–24].

### Participants & recruitment

#### Quantitative (cross-sectional)

A consecutive sample of children and adolescents was identified by clinicians who screened outpatient clinic lists and the electronic medical record for participants who met eligibility criteria. Included were children or adolescents with a confirmed diagnosis of CP, aged 5 to 18 years, with a dyskinetic or mixed dyskinetic/spastic motor type. Exclusion criteria were a major neurosurgical (i.e. deep brain stimulation, intrathecal baclofen implantation, selective dorsal rhizotomy) or orthopaedic procedure (i.e. bony or soft tissue surgery) within the previous 6 weeks or children and/or carers unable to speak English.

#### Qualitative (interviews)

Using data elicited from the larger quantitative cross-sectional study, we purposively selected participants with chronic pain (> 3 months) to invite to take part in qualitative interviews [7]. Purposive sampling ensured age groups (younger children and adolescents) and CP subtypes (mixed and dyskinetic) were adequately represented within the sample. Participants were selected who perceived impacts to their quality of life and were using pain treatments. Capacity to self-report was determined through mutual discussion between the researcher and carer which considered age, maturity, pain recall, intellectual functioning, and communication ability using the Communication Function Classification System (CFCS) Level. The CFCS (I-V) classifies everyday performance and effectiveness of communication in people with CP in terms of sending and receiving messages with a communication partner [25]. Participants classified within levels I-III were deemed capable of self-reporting.

### Data generation

#### Quantitative (cross-sectional study)

Following consent, a trained physiotherapist arranged a time to administer the pain survey, typically alongside an outpatient clinic appointment. The presence of a dyskinetic or mixed dyskinetic/spastic CP motor-type was confirmed using the Hypertonia Assessment Tool [26] and Cerebral Palsy Description Form of the Australian CP Register [27]. Demographics collected included age, gender, Gross Motor Function Classification System, CFCS, Manual Ability Classification System, intrathecal baclofen pump/deep brain stimulation, presence of seizures and gastrostomy, previous history of bony hip or spinal surgery, pain treatments and services accessed. A flexible approach to pain survey administration was adopted to accommodate the varying nature of communication amongst participants.

Quantitative outcomes were used to characterise the profile of pain across participants. Chronic pain was measured using the International Association for the Study of Pain definition of constant or recurrent pain over the previous 3 months [7]. Pain intensity using the Faces Pain Scale Revised (FPS-R) (1–10) measured over the previous 2 weeks, a psychometrically tested tool in other paediatric pain conditions [28, 29]. Painful body locations were measured using the Childhood Arthritis and Rheumatology Research Alliance Body diagram (21 defined regions) measured over the previous 2 weeks, a valid and reliable paediatric chronic pain measure [30]. Able participants marked the body regions of pain themselves; otherwise, the researcher marked body regions on their behalf. Quality of life was measured using the child or teen self-report versions of the Cerebral Palsy Quality of Life Questionnaire (CPQOL), which gives a 0–100 score for each domain and overall quality of life, with higher scores indicative of better quality of life [31].

#### Qualitative (interviews)

In-depth semi-structured interviews were conducted by a single interviewer (CM), typically at home or in a private location within the tertiary hospital depending on family preference. Interviews were video recorded with permission and transcribed verbatim, along with a detailed description of non-verbal aspects of their communication. Interviews ranged from 30 to 70 min, with extended interviews required for children requiring rest periods for fatigue or with slower paced communication. The interviewer (CM), an experienced physiotherapist trained in interview techniques and AAC use, was familiar with participants having previously administered the majority of the pain surveys. Having a prior relationship enabled the researcher (CM) to be more familiar with each child’s communication strategies, facilitate rapport and help put the participants at ease with the interview process. Carers were present during the interview and acted as communication partners as needed. A semi-structured interview guide of simple open ended questions with closed-ended prompts was created with the research question in mind and re-shaped in response to consumer feedback (Table 1). Before the interview, carers of participants were provided with an open-ended interview guide and encouraged to help participants prepare answers in an attempt to facilitate pain recall, minimize fatigue, and enhance comfort during the interview. Participants chose a preferred position and their most consistent method(s) of communication for interview. Carers were instructed to allow their child to answer as much as possible on their own. However, if a child deferred to their carer, additional effort was made to clarify that their carer’s interpretation was correct [18]. Each interview commenced by discussing the participant’s general medical care with both carer and child and by choosing a familiar word to describe their dyskinesia. Participants reviewed previously completed body diagrams and chose their most bothersome bodily pain location to discuss during the interview. Subsequent questioning explored the daily impact of pain and dyskinesia, including triggers, recognition, and treatments.

**Table 1:** Interview Guide

Question	
Can you tell us about the people who help look after you?	
How should we describe the *uncontrolled movements* in your body?	
What is it like to have *uncontrolled movements* in your body?	
Interviewer and participant review previously completed body diagram to clarify pain status and most bothersome body location of pain	
How does this pain get in the way of your life?	
How does pain make you feel on the inside?	
Do the people who look after you at school, home, and the hospital know about your pain?	
What things do you do when you are in pain?	
What things do other people do to help you?	
What would help you to look after your pain better in the future**?**	

### Data analysis

Quantitative and qualitative data were weighted equally and analysed separately, with triangulation and synthesis of data occurring in the interpretive phase.

#### Quantitative (cross-sectional study)

Data were analysed descriptively using STATA version 14.0 (Stata Corp, College Station, TX, USA). For chronicity and body locations, prevalence proportions and 95% confidence intervals (CI) were calculated. For demographic data and the FPS-R, frequencies and proportions were calculated, and for the CPQOL, means and standard deviations were calculated. Data was represented graphically where possible.

#### Qualitative (interviews)

Two authors (CM, JW) independently coded the data using IPA as outlined by Smith, Flowers, & Larkin (2009) [24]. CM is a doctoral student who was trained in qualitative data analysis techniques by JW, an experienced qualitative researcher. IPA proposes that researchers are trying to make sense of the participant’s world, while research participants are trying to make sense of their world within their words and descriptions [24]. It is idiographic, in that each case is analysed in full before moving onto the next case. The first step involved both authors immersing themselves in the data by reading and then re-reading transcripts, making initial comments and notes manually regarding content and meaning within transcript margins. After this process of initial noting, Nvivo 12.0 was used for the subsequent steps of analysis. Initial notes were developed into emergent interpretative themes, with exemplar quotes captured, and processes of abstraction used to search for connections between themes within each case. At this stage, the authors compared and discussed emergent themes and categories to confirm interpretations were firmly grounded in the data, and iteratively revised themes as necessary. Any disagreements were resolved by consensus, and as new emergent themes arose, transcripts were re-read and cross-checked for the emergent themes. After all cases had been analysed, both authors then searched for patterns and connections across cases. Sub themes were identified and refined into super-ordinate categories summarized in table form. Rigor within data collection and analysis was encouraged by having regular meetings between authors to peer de-brief and openly discuss and compare data analysis findings. Data trustworthiness was facilitated by using strategies such as data immersion and reflexive practice [32].

## Results

**Fig. 1:**
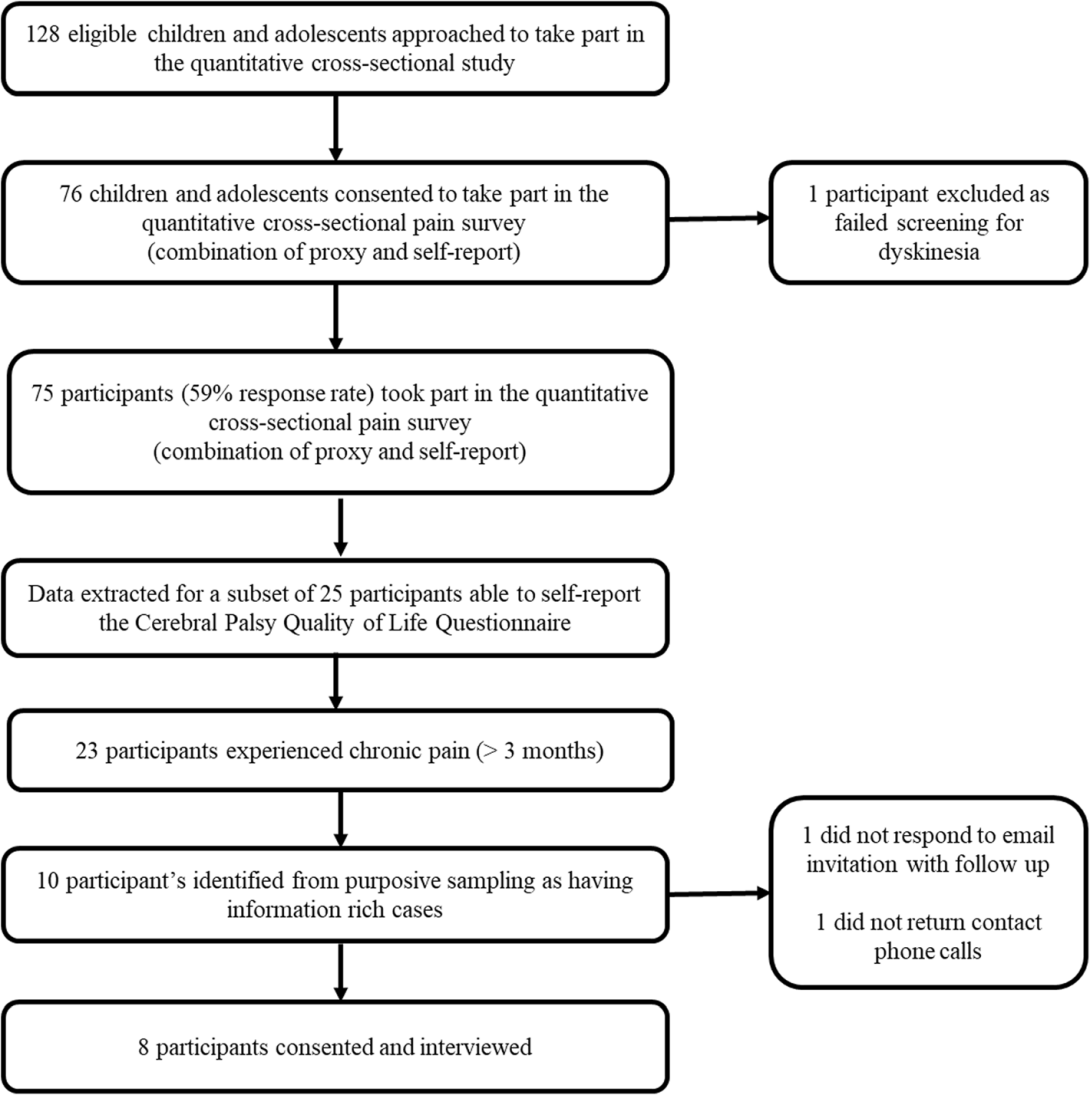
Study flow diagram of the convergent parallel mixed methods study

A subset of 25 children and adolescents completed the cross-sectional pain survey study, with ten identified by purposive sampling and eight subsequently consenting to take part in interviews. The overall study flow is described in Fig. 1.

### Quantitative

#### Demographics

For the 25 participants who took part in the cross-sectional (quantitative) study, mean age was 13.3 years (SD 3.6, 9 to 18 years), 15 were male, and 14 had a predominantly dyskinetic motor type. Full baseline participant demographics for this study are described in Table 2.

**Table 2:** Baseline demographics for the cross-sectional (quantitative study)

Baseline Measure	Total, ***n = 25***
Sex	
Male	15
Female	10
Mean (SD) age, y	
Range 9-18y	13.28 (3.17)
Age group, n	
≤ 12 years	10
> 12 years	15
Typology of CP	
Bilateral	21
Unilateral	4
Motor type	
Dyskinetic	14
Mixed	11
GMFCS, n (%)	
I	4
II	2
III	4
IV	9
V	6
MACS, n (%)	
I	2
II	4
III	9
IV	3
V	7
CFCS (I-V), n (%)	
I	13
II	6
III	6
IV	0
V	0
Invasive tone management	
(ITB/DBS), n (%)	3
Previous bony hip surgery, n (%)	10
Spinal fusion, n (%)	3
Seizures, n (%)	9
Gastrostomy	4

#### Pain and quality of life outcomes

**Fig. 2:**
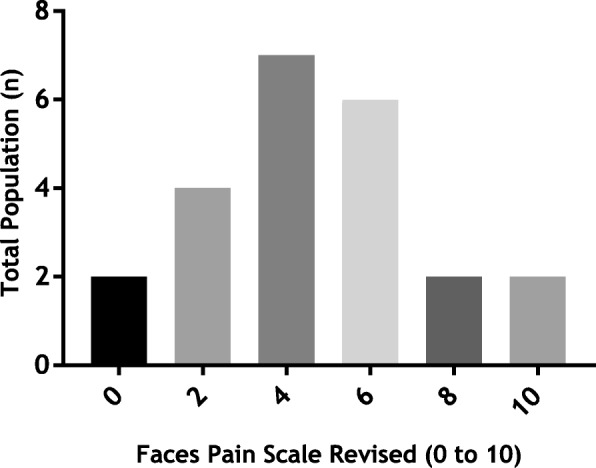
Pain intensity (*n* = 23) according to the Faces Pain Scale Revised over the previous 2 weeks

**Fig. 3:**
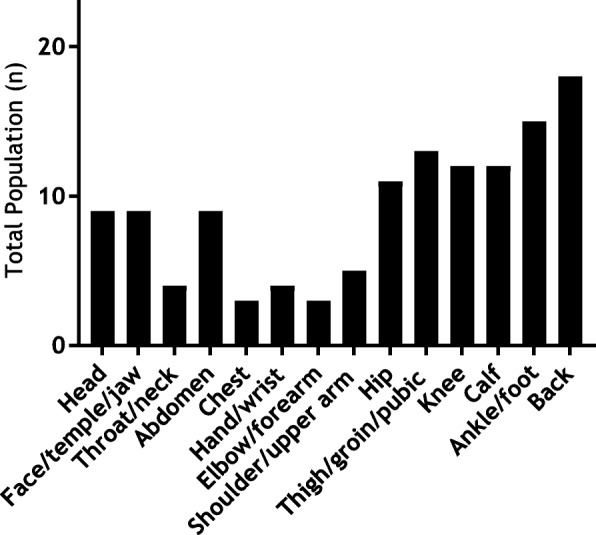
Body pain locations over the previous 2 weeks (*n* = 23)

Twenty-three of the 25 participants experienced chronic pain and in 13 this was of moderate intensity (4 to 6) according to the FPS-R (Fig. 2). Pain occurred in the lower limbs in 19 participants and in 6/21 (SD 5.8) body locations on average (Fig. 3).

**Fig. 4:**
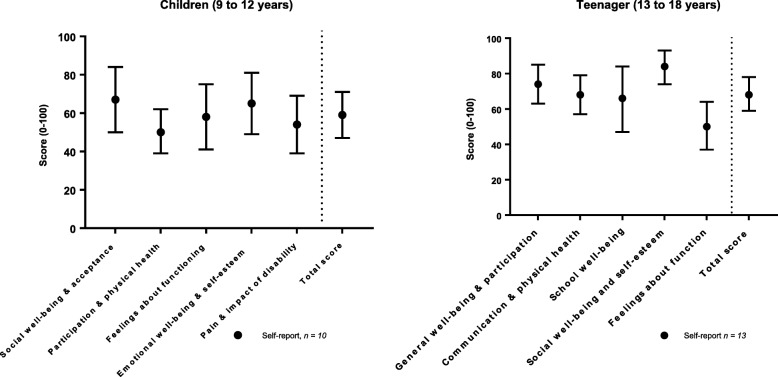
Cerebral Palsy Quality of Life Questionnaire domains and summary scores (mean and 95% confidence intervals) (*n* = 23)

Total CPQOL scores for children (9 to 12 years) were 58.94 (SD 16.2), with the participation & physical health domain scoring lowest at 50 (SD 23.9) and the social well-being and acceptance domain scoring highest at 67 (SD 15.5) (Fig. 4). The pain and impact of disability domain was positioned at the lower end of this range at 54 (SD 20.9). Total CPQOL scores for teenagers (12 to 18 years) were 68.32 (SD 16.2) (Fig. 3), with the feelings about function domain scoring lowest at 50 (SD 23.0) and social well-being and participation domain scoring highest at 84 (SD 15.5), while the communication and physical health domain was positioned in the middle of this range at 68 (SD 18.2).

### Qualitative

#### Demographics

For the 8 participants who took part in qualitative interviews, mean participant age was 11.6 years (SD 3.0, range 9 to 16 years), six were male, and four were predominantly dyskinetic. Three participants experienced mild to moderate pain not impacting activity levels, while the remaining five experienced moderate to severe pain impacting activity levels. Four children were able to verbalize, while the remaining used AAC. Three participants were classified as CFCS I, two as CFCS II, and three as CFCS III. Full baseline participant characteristics are described in Table 3, along with their pain conditions and management in Table 4. From the data, three main super ordinate themes emerged from subthemes described below and further summarized in Table 5.

**Table 3:** Baseline demographics for interviews

Child	Age (yrs)	Schooling	Motor type (mixed or dyskinetic)	Communication Methods	Child’s capability & disability			Previous bony hip or spinal surgery (Y or N)
					Gross Motor Function Classification System (I-V)	Communication Function Classification System (I-V)	Manual Ability Classification System (I-V)	
A	13–16	Special school	Mixed	Head nodding yes/no, hand gestures, finger pointing device	V	II	III	Y
B	13–16	Special School	Dyskinetic	Verbal yes/no, head switching using Dynavox	V	III	V	N
C	9–12	Mainstream	Mixed	Verbal	IV	I	III	N
D	13–16	Mix of special and mainstream	Dyskinetic	Visual scanning using PODD book and Yes/No card	V	III	V	N
E	9–12	Mainstream	Mixed	Verbal	IV	I	IV	Y
F	9–12	Mainstream	Dyskinetic	Verbal	IV	II	V	Y
G	9–12	Mainstream	Mixed	Verbal	III	I	III	Y
H	9–12	Mainstream	Dyskinetic	Visual scanning using PODD book	V	III	V	Y

*PODD* Pragmatic organizational dynamic display communication book, Tobii Dynavox Ltd. (Danderyd, Sweden)

**Table 4:** Baseline pain conditions and their management for interviewed participants

Child	Body locations (upper limbs, lower limbs, back) (n/21)^a^	Faces Pain Scale Revised (0 = 10)	Health Utilities Index - 3 prior 2 weeks (1–5)	Pain medications prior 3 months	Non-pharmacological pain treatments prior 3 months	Health professionals consulted regarding pain prior 3 months
A	Abdomen, back, lower limbs (6/21)	4	4	Oxycodone, gabapentin	Positioning, hydrotherapy	Paediatrician, orthopaedic surgeon
B	Face/temple/jaw, head, lower limbs (3/21)	6	2	Paracetamol, gabapentin	Hot pack, massage, distraction, rest	Paediatrician
C	Abdomen, lower limbs (3/21)	4	2	Paracetamol	Massage, positioning	Physiotherapist
D	Head, lower limbs, back, abdomen, face/jaw/temple (11/21)	4	3	Diazepam, paracetamol, ibuprofen	Massage, heat packs, cuddles, distraction, positioning	Physiotherapist, paediatrician
E	Lower limbs, throat/neck, 5/21	6	2	Paracetamol	Massage, stretching, exercises	None
F	Face/jaw/temple, chest, abdomen, groin/pubic area, back (9/21)	10	5	Paracetamol, ibuprofen, gabapentin, diazepam	Hydrotherapy, stretching, positioning, leg splints	Physiotherapist, paediatrician, surgeon, emergency room doctor, pain specialist
G	Back, head, lower limbs (6/21)	2	3	Paracetamol, ibuprofen	Stretching, icepacks, massage, rest, strengthening exercise, changing positions, activity modification	Physiotherapist, occupational therapist
H	Face/jaw/temple, chest, abdomen, groin/pubic, back, throat/neck, upper limbs, lower limbs (20/21)	8	2	Paracetamol, ibuprofen, midazolam	Feldenkrais techniques, chiropractor adjustments, massage, stretching, positioning	Paediatrician Feldenkrais therapist, physiotherapist, chiropractor

^a^Body locations of pain were measured according to the Child Arthritis and Rheumatology Research Alliance Body Diagram

**Table 5:** Super-ordinate categories and their sub themes

Super-ordinate category	Sub-theme
**Lives embedded with dyskinesia**	Lacking control Negative emotional responses towards living with CP and dyskinesia Normalization of dyskinesia within everyday lives Physical challenges of living with dyskinesia Self-reflecting to identify dyskinesia triggers
**Still learning strategies to manage their pain and dyskinesia**	Self-adopting or learning strategies from parents Seeking out ‘child friendly’ treatment strategies Problem solving pain Seeking comfort amongst family Exerting control over their environment
**The real world challenges of living with chronic pain**	Pain a usual part of life Negative emotional responses towards living with chronic pain Feelings of social isolation All-encompassing nature of severe pain Pushing through pain Hoping for a solution The way pain really feels Trust in caregivers

#### Lives embedded with dyskinesia

Participants emphasized the physical and emotional impacts of living with CP and dyskinesia, highlighting the variable nature of personal challenges and life contexts within which chronic pain was embedded. Some participants reported that the physical presence of the movement disorder had become a normal part of their everyday lives and they perceived little impact, even feeling *“comfortable [accepting of it]”* [Child H]. Child G reported, *“I would say nothing, I don’t notice them [dyskinetic movements]”.*

In contrast, other participants expressed more intense feelings towards experiences of living with the movement disorder characterised by annoyance, frustration, anger, and sadness. Such feelings were often closely linked to activity and role restrictions, compounded by experiences of living with disability. Child B perceived a lack of control over his life because of dyskinesia, reporting that he felt *“like a baby … …*. *like I can’t help my CP”*. He communicated the intensity of his emotions by using his maths times table and repeating his words to give them emphasis expressing, *“[I’m] really times 11 really really really really really times 11 angry”.* Child B’s anger towards living with CP and dyskinesia stemmed from feeling as though future aspirations such as *“jump out of plane”* were unachievable. Child D reported that her dyskinesia made her feel *“yuk”* and she found the physical presentation *“embarrassing”*. Child D was frustrated at not being able to use her hands as she wished, expressing *“need, grasp, can’t do myself”* and a wish to be able to *“draw”* more.

From an early age, participants reported that they, and their families, had used a range of lay language to describe and make sense of their dyskinesia such as: *“shaky movements”* [Child A], *“flapping like a chicken”* or *“contrary arm”* [Child B], *“blender body”* [Child D], *“jumpy movements”* [Child E], *“tricky body day”* [Child F], *“bad body day”* [Child F], and *“energetic movements”* [Child H]. These child-like expressions helped give context to dyskinesia experiences, allaying feelings of uncertainty or anxiety surrounding the sudden and unpredictable nature of their bodily movements. Many participants required prompts to self-reflect to identify possible triggers for their dyskinesia exacerbations, which were often more difficult to elicit in those who perceived dyskinesia to have little impact on their everyday lives. Identified activity triggers included concentrating and applying effort to schoolwork, playing sports, going to the toilet, and faster paced movements. Child E described her dyskinesia triggers, *“when I have got a lot of energy … .. just when I’m doing things, I would say physical [activities]”*. Other triggers for dyskinesia included being upset, stressed, scared, excited, anxious, and in pain. However participant descriptions of pain and dyskinesia relationships were inconsistent. Child D identified *“pain”* to trigger her dyskinesia and dyskinesia that was triggered by other factors to also cause her pain. Conversely, Child E did not identify pain to trigger her dyskinesia and dyskinesia triggered by other factors did not cause her pain reporting, *“it [dyskinesia] doesn’t hurt or anything”*.

#### Real-world challenges of chronic pain

Participants experienced chronic pain along a spectrum from those who experienced lesser pain to those experiencing a greater extent of pain intensity. For many, pain had been persistent throughout their childhood, Child C commenting, *“oh a long time, I can’t remember how long”*. All participants reported a range of different manifestations of childhood pain including comorbidities such as dyskinesia, constipation, reflux, headaches, and musculoskeletal issues. Two participants (Child A post spinal surgery, Child F post bony hip surgery) experienced severe and prolonged post-surgical pain, which left them physically debilitated waiting in hope for a medical solution. Some children (Child B, Child H) chose to discuss pain caused by seemingly less significant ailments such as mouth ulcers. Child B identifying his mouth pain to occur “*when I bite [my] tongue*”, creating a “*sharp*” sensation that was “*bloody sore*”. For Child B, his mouth ulcers had disrupted his favourite activity of eating for over a year and had been difficult to treat, making him feel “*angry*”.

In addition to emotions arising from living with dyskinesia, several participants expressed feelings of annoyance and frustration towards their persistent pain. *“Oh I think it makes me feel sort of frustrated that it’s [the pain is] happening and I can’t do something”* [Child C]. For some, physical restrictions caused by pain resulted in feelings of social isolation. Child A, who was unable to attend school because of his chronic pain, reported feeling *“lonely”*, *“bored”*, and *“sad”*. His feelings of social isolation were heightened by being unable to sit in his wheelchair for long enough to travel and visit highly valued relatives like his “*grandmother*” and “*cousin*”. His physical limitations further restricted his ability to take part in fun social activities like going to the movies with his family and friends. This left him feeling dependent due to having to wait for friends and family to visit him at home. Child D further reported feeling *“lonely”* at her mainstream school when in pain, feeling isolated and unsupported when her daily activities were not modified to accommodate changes in her pain condition.

Some participants reported pushing through their pain in an effort to maintain participation in valued activities such as socializing with friends, playing sports, going to school, and going out on family outings. *“Oddly … No it [pain] doesn’t actually really stop me from doing anything”* [Child C]. Child B also reported pushing through his mouth pain, expressing that he was still able to “*eat like a horse”*. One participant took pride in being determined reporting, *“I’m tougher than other children”* [Child C]. While another reported, *“If something’s hurting me, I probably won’t tell them … …*. *It’s not that I don’t want to, I just don’t”* [Child E]*.*

However, for some participants chronic pain was daily and debilitating. Child D reported her pain caused by dystonia felt like a *“crash with [a] rubbish truck”* while Child A described his ongoing back pain post spinal surgery to feel like his *“bones were breaking”*. Many participants were increasingly dependent on their family support network to accommodate their care and provide meaningful engagement. In response to being unable to participate in the playground, Child C reported, *“I just run into [sister’s name] and watch them play”.*

All participants expressed great confidence in their carers’ capacity to effectively pick up and respond to their pain. Indeed, one participant felt anxious and worried about not being in his carer’s presence and distrusting of others to provide effective care.

*Interviewer: What worries you about being at school on your own without your mum?*

*Child A: [Gestures towards back]*

*Communication partner for Child A: Your back. [Confirms child’s gesture]*

*Interviewer: Do you think they’d [school staff] be able to understand you if you needed to be moved?*

*Child A: [Head nod to indicate yes]*

*Interviewer: Do you think that they would know what to do?*

*Child A: [Head shakes to indicate no]*

#### Still learning strategies to manage their pain and dyskinesia

All participants acknowledged the lifelong reality of living with chronic pain, and demonstrated use of chronic pain management strategies that were ad hoc and learnt over time with their carer’s help. For example, Child F reported that his mum and dad had taught him how to manage his pain with little input from a health professional. Participants were able to problem solve the short-term management of a pain exacerbation, with carers facilitating management (e.g. passive stretching, massage), particularly for those with high levels of functional impairment.

Participants often spoke first about distraction strategies used to help when in pain such as music, television, YouTube, computer games, internet, jokes, iPads, pets, cuddles, toys, and food. For example, Child B suggested *“ice cream”* was helpful for his mouth pain caused by ulcers. While Child F suggested his favourite toy, watching the *“top ten funniest commercials”* on his iPad, and receiving *“cuddles”* from his parents were all helpful for his lower limb pain.

Participants valued treatments that provided them with periodic relief from pain, with little mention of exercise or activity-based approaches being used to manage persistent pain. Treatments identified included positioning, stretching, massage, heat/cold, medications, rest, and controlled breathing. Participants also valued having control over their body position and environment when in pain, finding it difficult to cope with unfamiliar noises and stimuli. Child H reported that at school he didn’t like to *“hear other noises”* when in pain, which meant making an excuse to escape to secluded areas like the *“toilet”.* In the toilet, he could both escape the noise and spend time in his preferred position of lying on his back on the change table. While at home he liked to be in communal environments like the *“lounge room”* where he could continue to engage with his family.

Participants also identified a range of helpful strategies for dyskinesia, some of which overlapped with their pain management including positioning, distraction, and controlled breathing. Child E described *“I just relax a bit and think less … … And then it [dyskinesia] just stops with that”*.

## Discussion

This study elicited key perspectives from children and adolescents with CP with the capacity to communicate their feelings and experiences of living with chronic pain and dyskinesia. This population is an unrepresented voice within current CP pain research, with the inclusion of AAC users providing an accurate reflection of all those able to self-report within the CP population. Within the study cohort of children with chronic pain, pain was often of moderate intensity, located over multiple body regions, and had varying influences over quality of life. Interviews highlighted the highly individual and varied nature of pain and dyskinesia experiences across this distinct cohort, ultimately reflecting the complexity of living with chronic pain and disability.

Despite high chronic pain prevalence being previously reported amongst children with CP and dyskinesia, no studies have explored qualitative perspectives of those able to self-report [33]. However a previous qualitative study has been undertaken of a broad CP cohort (across all subtypes) of adolescents and young adults with chronic pain able to verbalize their experiences [34]. Several common themes were identified between this study and ours relating to chronic pain’s all-encompassing nature, pushing through painful symptoms, impacts on role functioning and emotional well-being, reduced socialization, and family support roles. Study findings further add to this body of literature, by identifying specific themes relating to the impact of pain and dyskinesia interactions on daily living and across bio-psychosocial models. This new knowledge helps to improve understanding of specific pain issues facing children and adolescents with CP and dyskinesia within the broader CP population.

This study highlighted varying perspectives and impacts of living with chronic pain and dyskinesia across bio-psychosocial models. Participant’s identified individualized triggers for their dyskinesia, which were commonly framed by emotional, physical, and cognitive factors previously identified to drive dyskinesia exacerbations [4]. However this study uniquely described dyskinesia triggers with a practical and real world lens, not yet highlighted within current published literature. Across participants there were inconsistent pain and dyskinesia relationships, whereby dyskinesia was reported to form a pain source, and other pain sources reported to trigger dyskinesia, only in some participants. Such variability in clinical presentations highlights the need for flexible clinician approaches, that engage children and adolescents within pain assessment, so relevant information can be elicited to permit individualized therapy and enhance quality of life [4, 35]. The normalization of pain within children’s lives and some children failing to alert others of their pain issues, further reinforces the need for clinicians to pro-actively screen for pain as recommended by management guidelines in this area [36]. Pain screening has the potential to assist with earlier pain identification and management, a recognized need across the CP population [37, 38]. Engaging children and adolescents within pain screening and assessment is particularly important to elicit non-biased information, especially given child management priorities may differ from those of clinicians and carers. For example, some children chose to focus their interviews on relatively minor ailments which bothered them (e.g. mouth ulcers), rather than other more commonly recognized musculoskeletal issues. However eliciting pain-related information from those able to self-report is inherently complex given variable ability to self- report when using different pain tools, communication strategies, and experiencing differing levels of physical pain and fatigue. Ultimately, study findings reinforce the need to actively engage children within pain assessment, using communication partners as necessary, to determine clinically appropriate and personalized treatments aligning with child preferences.

Some children and adolescents reported strong emotional responses (e.g. anger and sadness) towards living with CP and dyskinesia and chronic pain. The presence of negative emotional responses reinforces the need to assess and treat psychological/emotional function as needed within chronic pain management. The need to support psychological/emotional function during chronic pain episodes, is further reinforced by findings that indicate children with dyskinetic CP experience increased social and emotional difficulties compared to typically developing children [39]. However, participants with chronic pain in this study did not discuss using psychological therapies, nor had they accessed psychological services in the 3 months prior to completing the cross-sectional pain study. Minimal use of psychological therapy was also reported by a recent Australian study of a broad CP cohort with chronic pain, highlighting possible gaps in service delivery in this area [33]. Despite children not formally using psychological interventions in this study, some did self-adopt ad hoc components of interventions such as mindfulness, including strategies such as controlled breathing and distraction, which they found helpful [40]. Use of psychological therapy (e.g. cognitive behavioural therapy) is considered vital to mitigating maladaptive chronic pain responses and for promoting effective self-management [35]. The importance of addressing such needs, was highlighted by a recent French study that reported children with CP to demonstrate less effective pain coping strategies compared to typically developing populations [41]. Findings of this study, highlight a need to actively assess and treat psychological/emotional function of children with CP and dyskinesia with chronic pain to ensure pain is effectively treated within bio-psychosocial models.

This study found that children and adolescents tended to discuss treatments that gave them short term pain relief, rather than graded exercise approaches to their management. Such approaches are likely to be integral to mitigating the negative sequelae of chronic pain such as reduced school attendance, social isolation, and altered role functioning [35]. It is difficult to determine whether graded exercises approaches were being implemented or if children had not been educated regarding these, with several children reported to be accessing exercise based therapies (e.g. hydrotherapy, physiotherapy) at baseline as reported by their carers (Table 3). This lack of discussion of exercise approaches may have been linked to reporting bias, whereby children and adolescents may have chosen to focus their interviews on the perceived interests of the interviewer. However strategies were adopted within this study to minimize potential bias and power imbalance between the researcher and participant (e.g. interviewing at home, open and flexible interview approach). Ultimately, discrepancies in participant understanding of exercise approaches to managing chronic pain with their daily lives, highlights a need for structured approaches to chronic pain education that empower knowledge and understanding of their condition.

The strength of this study lies in the capture of unique unbiased perspectives of children and adolescents able to self-report and from eliciting new information regarding the dual impact of dyskinesia and pain on everyday lives. The generalizability of findings from the quantitative cross-sectional study were limited due to small sample size and non-population sampling. In addition, some outcome measures had not been previously validated in CP and were chosen pragmatically based on the best available generic tools (i.e. the FPS-R and body diagram). Despite these limitations, quantitative findings were helpful in describing pain characteristics across the small cohort and served to complement the richness of qualitative findings. Homogenous sampling for qualitative interviews, as outlined by IPA, may have been limited by contextual differences in pain experiences between younger childhood (9–12 years) and adolescence (12 to 18 years) and across participants with differing levels of dyskinesia severity. However, a reasonably targeted sample was achieved considering the small and distinct nature of the subset of children with CP and dyskinesia able to self-report within the CP population. Despite several limitations, this study effectively highlighted the individuality of pain and dyskinesia experiences, with themes giving valuable insight into the variable nature of perspectives and experiences.

## Conclusions

Children and adolescents living with chronic pain and dyskinesia experienced multi-dimensional impacts within bio-psychosocial models. There is a need to directly educate children and adolescents about their chronic pain and for delivery of structured multi-modal therapies around pain including psychological interventions.

## Data Availability

Data is unable to be shared as it is against Human Research Ethics Committee approval and consent conditions.

## Abbreviations

AAC: Augmentative and Assistive Communication
CFCS: Communication Function Classification System
CP: Cerebral palsy
CPQOL: Cerebral Palsy Quality of Life Questionnaire
FPS-R: Faces Pain Scale Revised
